# Integrating community health representatives with health care systems: clinical outcomes among individuals with diabetes in Navajo Nation

**DOI:** 10.1186/s12939-019-1097-9

**Published:** 2019-11-27

**Authors:** Letizia Trevisi, John E. Orav, Sidney Atwood, Christian Brown, Cameron Curley, Caroline King, Olivia Muskett, Hannah Sehn, Katrina A. Nelson, Mae-Gilene Begay, Sonya S. Shin

**Affiliations:** 1 Department of Global Health and Social Medicine, Harvard Medical School, Boston, MA USA; 2 Department of Medicine, Brigham and Women’s Hospital, Boston, MA USA; 3 Division of Global Health Equity, Brigham and Women’s Hospital, Boston, MA USA; 4 School of Medicine, Oregon Health and Science University, Portland, OR USA; 5 Partners in Health, Boston, MA USA; 6 Navajo Department of Health, Window Rock, Window Rock, AZ USA

**Keywords:** Chronic disease, Community health workers, Diabetes mellitus, Education and behavioral intervention, Navajo

## Abstract

**Background:**

We studied the impact of Community Outreach and Patient Empowerment (COPE) intervention to support Community Health Representatives (CHR) on the clinical outcomes of patients living with diabetes in the Navajo Nation extending into the States of Arizona, Utah, and New Mexico. The COPE intervention integrated CHRs into healthcare teams by providing a structured approach to referrals and home visits.

**Methods:**

We abstracted routine clinical data from the Indian Health Service’s information system on individuals with diabetes mellitus seen at participating clinical sites from 2010 to 2014. We matched 173 COPE participants to 2880 patients with similar demographic and clinical characteristics who had not participated in COPE. We compared the changes in clinical outcomes between the two groups using linear mixed models.

**Results:**

Over the four years of the study, COPE patients had greater improvements in glycosylated hemoglobin (− 0.56%) than non-COPE participants (+ 0.07%) for a difference in differences of 0.63% (95% confidence interval (CI): 0.50, 0.76). Low-density lipoprotein fell more steeply in the COPE group (− 10.58 mg/dl) compared to the non-COPE group (− 3.18 mg/dl) for a difference in differences of 7.40 mg/dl (95%CI: 2.00, 12.80). Systolic blood pressure increased slightly more among COPE (2.06 mmHg) than non-COPE patients (0.61 mmHg). We noted no significant change for body mass index in either group.

**Conclusion:**

Structured outreach by Community Health Representatives as part of an integrated care team was associated with improved glycemic and lipid levels in the target Navajo population.

**Trial registration:**

Trial registration: NCT03326206. Registered 31 October 2017 - Retrospectively registered, https://clinicaltrials.gov/ct2/show/study/NCT03326206.

## Background

Diabetes mellitus (DM) is a prevalent chronic condition in many Native communities. Cardiovascular disease and type 2 DM (T2DM) are the 3rd and 4th leading causes of death in the Navajo Nation, respectively [1]. Mortality due to diabetes is almost twice as high among American Indian and Alaska Native populations than in the general population in the United States [2]. The cost of T2DM care among Native populations is considerable, estimated in one Indian Health Service (IHS) facility to account for 37% of all adult treatment expenses [3]. Lack of access to healthcare services and healthy food contribute significantly to the burden of diabetes [4] as well as other chronic health disparities affecting rural Native communities. Some scholars have argued that the rising rate of T2DM over the past few decades in Native communities correlates with the historical imposition of U.S. federal policies that have eroded local indigenous food systems and traditional lifestyles and diets [5].

Despite these structural challenges, unique strengths exist within tribal health care systems. One such resource is the Community Health Representative (CHR) Program which is instrumental in supporting and promoting health by addressing the social determinants of health at the community level. The CHR is a national cadre of well-trained, medically-guided, Native community-based health care providers who may include traditional Native concepts in their work [6].

The Community Outreach and Patient Empowerment (COPE) project was launched in 2010 in collaboration with the Navajo Nation CHR Program to improve health outcomes by supporting CHR outreach to individuals living with chronic diseases. The purpose of the intervention is to improve management of T2DM and comorbidities in order to reduce future cardiovascular risk [7, 8]. As a programmatic intervention, COPE focuses on increasing referrals of high-risk patients to Navajo Nation CHRs, supporting CHRs through training and standardized health promotion materials, and strengthening care coordination between CHRs and clinic-based providers. To date, this program has enrolled more than 650 patients in the Navajo Nation.

The purpose of this study is to measure the impact of the COPE program on the clinical outcomes of individuals living with T2DM in the Navajo Nation, who were referred to the COPE program by providers or CHRs themselves. The main outcome assessed is the change in glycosylated hemoglobin (HbA1c) averaged over the 24 months prior to enrollment in COPE and the 24 months after enrollment.

## Methods

### Study setting and population

The Navajo Nation includes 332,129 members and covers 27,400 square miles (71,000 km^2^) in parts of Arizona, Utah, and New Mexico [9]. It is organized into 110 Chapters, which are local government entities similar to counties or townships. The healthcare services on Navajo Nation are delivered in two different ways: through the Navajo Area Indian Health Service, one of 12 regional administrative units of the national IHS system, and through tribally-contracted health programs. The Navajo Area Indian Health Service operates in 5 hospitals, 7 health centers, and 15 clinics. The majority of population served by the Navajo Area IHS lives on the rural reservation. Along with its vast geography and limited infrastructure (with 78% of public roads still unpaved), low incomes and low population density discourage retail food sources on the reservation. Currently, an estimated 25,000 Navajo individuals are living with T2DM, and another 75,000 have pre-DM, encompassing 49.6% of the adult population [10]. The paucity of health facilities and high turnover of healthcare workers, combined with efforts to incorporate public health services into tribal healthcare, led to the introduction of the CHR program.

### Role of CHRs in Navajo Nation

The Navajo Nation employs nearly 100 CHRs to connect health facilities and medical providers to communities and help community members navigate these complex health systems by facilitating access to healthcare services, improving the quality of healthcare delivery, and offering community-based outreach for patients, families, and communities. CHRs are trained as Certified Nursing Assistants and are required to speak Navajo. Each CHR is assigned to one or more specific Chapter, depending on the size of the Chapters, vacancies, and the number of clients who need close monitoring. CHRs cover an average number of two Chapters, and have a caseload that ranges from 30 to 50 clients. While CHRs are available to everyone in their communities, they prioritize outreach to clients with chronic health conditions (e.g. T2DM, heart disease) and/or risk factors associated with social determinants of health based on their assessment of each client’s social support and living conditions. CHRs conduct home visits to these clients to check vital signs, assess medication adherence, monitor for any urgent health issues, and answer any questions that the patient might have. As Certified Nursing Assistants, CHRs are qualified to monitor vital signs and measure blood glucose levels using finger stick testing. CHRs also assist with many social issues by referring clients to programs that improve unsafe housing (e.g. adding a bathroom if there’s only an outhouse, wheelchair ramp), referring clients to food assistance programs, and coaching patients on how to arrange transportation to medical appointments.

### Program intervention

COPE is a multi-level intervention aimed at linking clinic-based providers, CHRs, and patients. The program is comprised of three inter-related strategies designed to strengthen the existing community outreach and linkage to clinic-based care [11].

1. *Enabling patient referral to the Navajo Nation Community Health Representative Program*. Providers are asked to refer patients at increased risk of chronic disease complications. The provider discusses enrollment with the patient and if the patient agrees, complete a referral to the CHR program, either through the IHS electronic health record or using a standard IHS paper referral form. CHRs may also refer clients identified through home visits, and clients may self-refer (or be referred by their family members) to the program (Table 1). Because CHRs provide services exclusively to individuals living on the reservation, patients enrolled in the program typically face significant geographic barriers to access healthcare and healthy food, and limited infrastructure such as utilities and paved roads [Table 1].
2. *Supporting community-based patient accompaniment*. The COPE team routinely provides additional training to CHRs in evidence-based behavior change strategies, such as Motivational Interviewing, patient-identified SMART goals (i.e. Specific, Measurable, Attainable, Relevant, Time bound), and delivers structured teaching materials in the form of flipcharts. COPE has developed culturally-informed flipcharts to cover specific health topics (e.g. cholesterol, diabetes foot care, physical activity) following the format of the 5A (Ask, Assess, Advise, Assist, Arrange follow-up) brief counseling intervention [12, 13]. Upon referral, CHRs make regular home visits to COPE participants, generally once or twice a month.
3. *Community-clinical linkages*. The COPE team works with CHRs and providers to increase bi-directional communication about patient management. COPE team works with providers to create access for CHRs on Electronic Health Records, initiate case management, create templates for electronic CHR referrals, and orient providers on CHRs’ work and the COPE intervention.

**Table 1:** COPE intervention additional components, Navajo Nation, United States, 2010–2014

Program Components	Before COPE collaboration	Introduced by COPE collaboration
Patient outreach	Home visits by CHRs without established frequency.	“COPE clients” receive home visits at least monthly and tracked as high-risk client.
	Each CHR prepares his/her own health education materials resulting in inconsistent health coaching.	CHRs deliver standardized coaching materials that have been vetted by local providers and ensure goal setting at each session.
	Vital signs monitored inconsistently, CHRs lack oximeters, multiple size blood pressure cuffs, or glucometer training / supplies.	Vital signs monitored; all CHRs equipped with oximeters, multiple size blood pressure cuffs, glucometer training / supplies.
CHR Training	CHRs receive training on health topics when available.	Monthly training sessions to CHRs on health topics taught by local providers to build CHR-provider relationship.
	CHRs do not receive training on motivational interviewing, self-care, goal setting.	CHRs receive training on motivational interviewing, self-care, goal setting delivered by Navajo-speaking trainers.
	Not competency assessments of CHR or trainer knowledge / proficiency.	Competency assessments administered at each training to assess CHR and trainer knowledge / proficiency.
	CHR supervisors receive training when available.	CHR supervisors receive monthly trainings in team building, supervision and leadership, quality improvement, and wellness / self-care.
Community-clinical linkages	CHRs work with Public Health Nurses to evaluate clients together and establish care plans; however, CHRs rarely coordinate care with other healthcare providers.	Increased bi-directional communication and care coordination through planning conjunct meetings, orientation of new clinical staff, provider-led CHR trainings, joint home visits, and conjunct case management.
	No access to Electronic Health Records for CHRs.	CHRs are able to gain access to Electronic Health Records to document home visits and obtain client information.
	Patients rarely referred by providers to CHRs; primarily identified by CHRs themselves.	COPE helped to increase the awareness of the CHR program with presentations in hospitals. Referral system established and increased referrals by providers to CHR Program.

*COPE* Community Outreach and Patient Empowerment, *CHR* Community Health Representative

All program participants receive routine standard of care in addition to the COPE intervention.

The COPE team was careful to allow CHRs to have flexibility in how they chose to deliver the intervention to their clients. The components that were consistently delivered to all COPE patients were: 1) health education using flipcharts at least one module monthly, 2) vital signs including blood glucose checks (if patient allowed) at each home visit, 3) communication with provider regarding any urgent or pressing clinical issues. Since the COPE team has developed the flipcharts and works with local providers to deliver training to the CHRs, the cost of the intervention maintenance is minimal, requiring ongoing time contributed by providers to train and ensure that flipcharts are updated (Table 1).

### Study design and outcomes

This quasi-experimental study was designed to evaluate the impact of the COPE intervention in patients living with T2DM by comparing clinical endpoints among DM patients receiving COPE and a comparable group that did not receive the intervention. Using routine clinical data from the IHS’s information system in six federal sub-regions, we identified patients enrolled in the COPE program who had an ICD-10 diagnosis of T2DM and received care at one of the participating study facilities. We abstracted laboratory test results, vital signs, medications, and health utilization data from 2009 to 2015 for these patients from the Resource and Patient Management System (RPMS), an electronic health record used by the majority of IHS facilities for routine clinical care. Of note, sociodemographic characteristics, such as educational level or income, were not available in routine medical records. Next, we identified non-COPE patients and included into the study those that could be matched to individuals in the intervention group on age (± 5 years), gender, primary health facility, and baseline hemoglobin HbA1c (± 1 point) and systolic blood pressure (± 10 mmHg) where baseline refers to a time point three months or less prior to the date of the COPE patient’s enrollment in the program. We assessed the difference in differences for the mean hemoglobin HbA1c during the 24 months prior to enrollment in COPE and the 24 months after enrollment. We also assessed changes in low-density lipoprotein (LDL), systolic blood pressure (SBP), and body mass index (BMI) for the subsets of patients with baseline essential hypertension, diagnoses of dyslipidemia, and a body mass index > 30, respectively.

To adjust for potential confounders, we collected data on the following patient characteristics: age (continuous), gender (male/female), preferred language (English, Navajo and other), whether the patient had a primary care physician; essential hypertension, major depression disorder, alcohol abuse, dyslipidemia, and major cardiovascular disease. We considered a participant to have major cardiovascular disease if he/she had one of the following diagnoses: acute myocardial infarction, coronary artery bypass surgery, coronary angioplasty, peripheral arterial disease, abdominal aortic aneurysm, carotid artery disease, cerebrovascular disease. We assessed the quality of clinical monitoring in COPE and non-COPE patients using a binary indicator that indicated whether HbA1c, LDL, and SBP had all been measured at least once between 12 and 24 months after enrollment. For this analysis we included all individuals with a pre-enrollment HbA1c value up to 24 months prior to enrollment, generating matched non-COPE to COPE participants using the same baseline variables described above.

### Statistical approach

We used mixed linear regression models to assess differences over time in the continuous outcome values and logistic regression to assess clinical monitoring, adjusting for the baseline outcome as averaged over the 2 years preceding the enrollment date and including a random effect term for patient and facility (Additional file 1 Appendix). We also included a random effect term for each matching set of COPE and non-COPE patients. We performed sensitivity analyses adjusting for the health facility effect as a fixed rather than random effect, and we evaluated the clinical outcomes using a narrower time-window: 12 months pre- and 12 months post-enrollment period (rather 24 months). All the analyses were carried out using SAS 9.3 (SAS Institute, Cary NC).

### Study registration

Our study design is observational and does not involve prospective assignment to an intervention. Patients were referred or not referred to COPE outreach by their provider or CHRs themselves as part of routine care, before the observational study began. For this reason, we do not consider this study a clinical trial. However, PCORI requires all studies funded by their organization must be registered in a study database; based on researching the different study registries, we felt that clinicaltrials.gov would be the most suitable. For this reason, the study was registered in clinicaltrials.gov after enrollment began.

## Results

### Study cohort and baseline characteristics

From December 2010 to August 2014, we identified 173 individuals COPE clients and 2880 non-COPE patients who met our inclusion criteria. The ratio of COPE to non-COPE participants varied depending on the number of matches; the median number of non-COPE patients matched to each COPE patient was 11 [range: 1–85].

As shown in Table 2, COPE and non-COPE patients were similar in most of patient characteristics and clinical diagnoses, except for primary language, and dyslipidemia. Among COPE clients, 58% reported Native American as their primary language compared to 41% of the non-COPE group. The prevalence of dyslipidemia at baseline was 46% among COPE patients and 59% among non-COPE patients. [Table 2] The baseline HbA1c LSM was 8.39% among COPE patients, and 8.23% in the comparison group, the baseline LDL LSM among COPE was 95.70 mg/dl versus 95.52 mg/dl of non-COPE, the baseline SBP LSM was 132.46 mmHg in the intervention group and 131.45 mmHg in the matched group, and the baseline BMI LSM was 36.57 kg/m^2^ and 35.88 kg/m^2^ in the COPE and in non-COPE, respectively (Table 3). The median number of HbA1c measurements pre-enrollment per subject was eight in both groups; the median number of post-enrollment measurements was seven for the COPE patients and eight for the non-COPE patients [Table 3].

**Table 2:** Baseline characteristics of COPE participants versus non-COPE participants, Navajo Nation, United States, 2010–2014

Characteristic	COPE	non-COPE	*p*-value
	n (%)	n (%)	
Total number of participants	173	2880	
Patient characteristics			
Age			
25–40 y	7(4.05)	46(1.60)	0.003
41–55 y	33(19.08)	610(21.18)	
56–70 y	74(42.77)	1436(49.86)	
71–85 y	54(31.21)	764(26.53)	
= > 86 y	5(2.89)	24(0.83)	
Gender			
Male	65(37.57)	901(31.28)	0.084
Female	108(62.43)	1979(68.72)	
Preferred Language			
Native American	100(57.80)	1178(40.90)	<.0001
English	72(41.62)	1700(59.03)	
Missing	1(0.58)	2(0.07)	
Primary Care Physician			
Yes	146(84.39)	2577(89.48)	0.036
No	27(15.61)	303(10.52)	
Clinical Diagnoses			
Essential Hypertension			
Yes	113(65.32)	1966(68.26)	0.400
No	60(34.68)	909(31.56)	
Missing		5(0.17)	
Major Depression Disorder			
Yes	25(14.45)	282(9.79)	0.049
No	148(85.55)	2593(90.03)	
Missing		5(0.17)	
Alcohol abuse			
Yes	11(6.36)	77(2.67)	0.015
No	162(93.64)	2798(97.15)	
Missing		5(0.17)	
Major Cardiovascular Disease			
Yes	123(71.10)	2050(71.18)	0.954
No	50(28.90)	825(28.65)	
Missing		5(0.17)	
Dyslipidemia			
Yes	80(46.24)	1688(58.61)	0.001
No	93(53.76)	1187(41.22)	
Missing		5(0.17)	

*COPE* Community Outreach and Patient Empowerment, *y* years, *HbA1c* glycosylated hemoglobin

Evaluation at the closest available HbA1c measure before the enrollment date

**Table 3:** Clinical outcomes comparisons at 24 months, Navajo Nation, United States, 2010–2014

	Intervention		Crude Model ^a^		Adjusted Model ^b^	
			Difference post VS pre intervention	95%CI	Difference post VS pre intervention	95%CI
	PRE	POST	HbA1c			
COPE	*N* = 173	*N* = 163				
	LSM (SE)	LSM (SE)				
	8.39 (0.05)	7.82 (0.06)	−0.57	−0.70, −0.45	−0.56	−0.69, −0.44
non-COPE	*N* = 2879	*N* = 2738				
	LSM (SE)	LSM (SE)				
	8.23 (0.01)	8.29 (0.01)	0.06	0.03, 0.09	0.07	0.04, 0.10
			*P* interaction	< 0.0001	*P* interaction	< 0.0001
	PRE	POST	LDL			
COPE	*N* = 72	*N* = 73				
	LSM (SE)	LSM (SE)				
	95.70 (3.85)	83.92 (3.87)	−11.79	−17.04, −6.53	−10.58	−15.85, −5.31
non-COPE	*N* = 1596	*N* = 1499				
	LSM (SE)	LSM (SE)				
	95.52 (2.13)	91.18 (2.15)	−4.33	−5.54, −3.13	−3.18	−4.41, −1.95
			*P* interaction	0.007	*P* interaction	0.007
	PRE	POST	SBP			
COPE	*N* = 113	N = 113				
	LSM (SE)	LSM (SE)				
	132.46 (0.97)	134.86 (0.97)	2.40	1.45, 3.35	2.06	1.10, 3.02
non-COPE	*N* = 1966	*N* = 1964				
	LSM (SE)	LSM (SE)				
	131.45 (0.36)	132.43 (0.36)	0.98	0.74, 1.23	0.61	0.35, 0.87
			*P* interaction	0.005	*P* interaction	0.004
	PRE	POST	BMI			
COPE	*N* = 59	N = 59				
	LSM (SE)	LSM (SE)				
	36.57 (0.71)	36.06 (0.71)	−0.51	−0.90, −0.12	−0.27	−0.67, 0.12
non-COPE	*N* = 1196	*N* = 1182				
	LSM (SE)	LSM (SE)				
	35.88 (0.16)	35.31 (0.16)	−0.57	−0.67, − 0.47	− 0.34	− 0.45, − 0.22
			*P* interaction	0.78	*P* interaction	0.75

*COPE* Community Outreach and Patient Empowerment, *HbA1c* glycosylated hemoglobin, *LDL* low-density lipoprotein (mg/dl), *SBP* systolic blood pressure (mmHg), *BMI* body mass index (kg/m^2^), *LSM* Least Squares Means, *SE* Standard Error, *CI* confidence interval, *P interaction* p-value of the interaction for the β of the term intervention(0/1)*time (pre/post)

^a^ Model adjusted for the pre-2 years’ means

^b^ Adjusted model for pre-2 years’ means, age (years; continuous), gender (male/female), preferred language (English, Navajo and other), primary care physician (yes/no); essential hypertension (yes/no), major depression disorder (yes/no), alcohol abuse (yes/no), dyslipidemia (yes/no), and major cardiovascular disease (yes/no)

### Study outcomes

Compared to mean adjusted HbA1c in the 2 years prior to COPE enrollment, mean HbA1c in the two years after enrollment decreased by − 0.56% (95% confidence interval (CI): − 0.69, − 0.44) among COPE patients and increased by + 0.07% (95%CI: 0.04, 0.10) among the matched non-COPE patients for a difference in differences of 0.63% (95CI%: 0.50, 0.76) (Table 3).

This change in HbA1c among COPE versus non-COPE patients differed significantly in both non-adjusted and adjusted models. The adjusted change in LDL among COPE patients was − 10.58 mg/dl, (95%CI: − 15.85, − 5.31) compared to − 3.18 mg/dl (95%CI: − 4.41, − 1.95) among the non-COPE patients for a difference in differences of 7.40 (95%CI: 2.00, 12.80). Systolic blood pressure increased in both groups, by 2.06 mmHg (95%CI: 1.10, 3.02) in the intervention group and 0.61 mmHg (95%CI: 0.35, 0.87) in the non-COPE group with a difference in differences of − 1.45 (95%CI: 0.46, 2.43). Changes in body mass index between the two groups were not significant (− 0.27 kg/m^2^ versus − 0.34 kg/m^2^, with a difference in differences of − 0.07, 95%CI: − 0.34, 0.47). Results were consistent when we further adjusted the main models for a site-specific variable. In sensitivity analyses using one-year pre-post time windows, findings were consistent with the two-year results in terms of magnitude and direction for all the four clinical outcomes (Additional file 1 Table S1).

As shown in Tables 4, 48.92% of the intervention group and 52.89% of the non-intervention group received “acceptable” disease monitoring between 12 and 24 months after enrollment. (adjusted odds ratio (OR) = 0.93, 95%CI: 0.68, 1.26) [Table 4].

**Table 4:** Logistic models for “disease monitoring” outcome, Navajo Nation, United States, 2010–2014

Rate of received “acceptable” disease monitoring	Intervention	OR	95% CI
48.92% in COPE	COPE vs non-COPE ^a^	0.85	0.63, 1.15
52.89% in non-COPE	COPE vs non-COPE ^b^	0.93	0.68, 1.26

*COPE* Community Outreach and Patient Empowerment, *OR* Odds ratio, *CI* confidence interval

^a^ Model adjusted for baseline monitoring disease (indicator variable that takes value 1 if HbA1c, LDL, and SBP, are all measured at least once in the 12 months before the enrollment date, and 0 otherwise)

^b^ Adjusted model for baseline monitoring disease, age (years; continuous), gender (male/female), preferred language (English, Navajo and other), primary care physician (yes/no); essential hypertension (yes/no), major depression disorder (yes/no), alcohol abuse (yes/no), dyslipidemia (yes/no), and major cardiovascular disease (yes/no)

Models evaluate the probability of having glycosylated hemoglobin (HbA1c), low-density lipoprotein (LDL), and systolic blood pressure (SBP), all been measured at least once between 12 and 24 months after the enrollment date

### Discussion

Participation in COPE among individuals living with T2DM was associated with improvements in HbA1c at 12 and 24 months and in LDL at 24 months. It is notable that changes in our cohort were sustained at 24 months, an outcome that is not usually assessed in evaluations of impact. The difference noted in HbA1c between the intervention and non-intervention groups over time reflects a clinically meaningful change which surpassed the average responses observed in T2DM self-management education programs described in a recent meta-analysis [14]. We also detected a significant reduction in LDL among COPE patients compared to non-COPE patients who had a baseline diagnosis of dyslipidemia. We observed a slight increase in blood pressure for both COPE and non-COPE patients. Lastly, we did not see improved changes in body mass index, or monitoring of standard clinical measures related to cardiovascular risk due to COPE participation.

Our results are consistent with those observed in the most effective community-based DM interventions reported in a systematic review of nutrition-based interventions among American Indian and other indigenous populations [3]. Our observed decline of HbA1c (− 0.42% versus + 0.04% and − 0.56% versus + 0.07% respectively at 1 and 2 years) was similar to the mean HbA1c changes in three studies that compared people receiving versus not receiving the intervention (ranging from + 0.38% versus − 0.46, + 0.4% versus + 1.2%, to − 0.8% versus + 0.3%) [15–17]. The American Diabetes Association cites 0.5% HbA1c as a clinically significant change [18]. For context, a 0.5% increase in HbA1c is associated with 9% higher odds of myocardial infarction [19], and an HbA1c reduction from 8.3 to 8.0% has been shown to significantly reduce the risk of retinopathy over an approximate 10-year span [20]. Furthermore, for our population of predominantly elder individuals, the average HbA1c achieved of 7.82 at 24 months likely reflect optimal control based on recommendations by the American College of Physicians [21]. The reduction of LDL by − 10.58 mg/dl observed in this study is comparable to decline in LDL levels of − 5.29 mg/dl noted by Moore et al. in a large-scale, intensive intervention among American Indian and Alaska Native populations with T2DM [22]. The 12% drop in LDL that we observed in our cohort confers meaningful clinical benefit, including a 19% relative reduction in the risk of nonfatal myocardial infarction or death due to coronary health disease [23, 24]. In contrast to these results, both COPE and non-COPE participants experienced a modest increase in SBP during the 2-year follow-up, with a small but statistically significant increase among COPE patients (+ 2.06 versus + 0.61 mmHg). We believe this increase has minimal clinical significance, considering the mean changes observed in clinical trials aimed at improving hypertension through CHW interventions, which ranged from − 6.5 mmHg versus − 2.7 mmHg, − 7.5 mmHg versus + 3.4 mmHg, to − 10.8 mmHg versus − 5.8 mmHg among people receiving versus people not receiving the intervention [25–27]. Neither COPE nor non-COPE participants experienced improvements in BMI.

Our findings are consistent with the growing evidence that supports the integration of community health workers (CHWs) on clinical care team, especially in underserved groups (African-American, Hispanic, or groups classified as low-income) [28]. Other studies have documented a significant impact of CHW-based interventions on non-communicable chronic diseases in the US, including improvements in cholesterol and blood pressure [29], as well as in reducing costs and preventable healthcare utilization [30]. A recent systematic review of CHW intervention for DM found a range of improved outputs and outcomes including knowledge about DM, diabetes self-care behaviors, clinical outcomes, and healthcare utilization and costs especially in Hispanic and non-Hispanic Black populations [31].

We note some limitations of our study. Firstly, while the use of routine clinical data provided a unique opportunity to compare COPE participants with thousands of individuals with comparable clinical indicators, not all the clinical outcomes were consistently measured and other variables related to sociodemographic characteristics and patient behavior were unavailable and could not be assessed as potential confounders or mediators. However we speculate that even if sociodemographic and behavioral differences exist between COPE and non-COPE patients, they would be likely skewed toward greater vulnerability among the intervention group. Second, this study was limited to individuals living with T2DM who were seen at a participating facility. Thus, findings may not be representative of DM patients who were not engaged in healthcare at all or seen at other facilities. We also note that we could have underestimated the impact of our intervention if there were positive spillovers effects within our sites, since the same CHRs also see non-COPE clients in addition to those enrolled in COPE. However, we estimate that the non-COPE patients seen by CHRs would only represent a small proportion of the enter non-intervention group (< 5%), unlike case of “study contamination” would likely bias toward the null.

## Conclusion

In a programmatic intervention carried out in a Native community, we observed improved diabetes outcomes, sustained over two years, among patients receiving structured CHW outreach. Our program findings support the growing evidence that CHW are effective in reducing chronic disease disparities, particularly when, CHWs are an integrated part of the healthcare. Given the prevalence of diabetes among underserved populations and the literature to support the role of community health workers in reducing health disparities [32], the findings of this study provide further evidence to support expansion of the CHW workforce as uniquely valuable healthcare professionals and widespread integration of CHW in U.S. healthcare systems. Future work will focus on understanding health utilization patterns of COPE patients, understanding the impact of COPE on patient-reported outcomes, and identifying patient characteristics that predict treatment response.

## Supplementary information

**Additional file 1: Table S1.** and **Appendix.** Table S1 reports the results for clinical outcomes comparisons at 12 months; the Appendix reports the details of the linear mixed models.

## Data Availability

The datasets generated and/or analyzed during the current study are not publicly available due to Navajo Human Research Review Board (NHRRB) policy that all research data collected in Navajo Nation belong to the Navajo Nation; they are available from the NHRRB on reasonable request.

## Abbreviations

CI: confidence interval
LSM: Least Square Mean
OR: odds ratio
