# Supplementary material for: Integrating community health representatives with health care systems: clinical outcomes among individuals with diabetes in Navajo Nation
Source: Int J Equity Health. 2019 Nov 27;18:183. doi: 10.1186/s12939-019-1097-9 (PMC6880375; doi:10.1186/s12939-019-1097-9)
Supplement: Supplementary file 1 — Additional file 1: Table S1. and Appendix. Table S1 reports the results for clinical outcomes comparisons at 12 months; the Appendix reports the details of the linear mixed models. [file 12939_2019_1097_MOESM1_ESM.docx]

**SUPPLEMENTAL MATERIALS**

**Table S1.**  Clinical Outcomes Comparisons at 12 months, Navajo Nation, United States, 2010-2014.

|  |  |  | Crude Model ^a^ | | Adjusted Model ^b^ | |
| --- | --- | --- | --- | --- | --- | --- |
|  | Intervention |  | Difference post VS pre intervention | 95%CI | Difference post VS pre intervention | 95%CI |
|  | **PRE** | **POST** | **HbA1c** | | | |
| **COPE** | N=173 | N=148 |  |  |  |  |
|  | LSM (SE) | LSM (SE) |  |  |  |  |
|  | 8.36 (0.06) | 7.93 (0.06) | -0.42 | -0.57, -0.27 | -0.42 | -0.57, -0.27 |
|  |  |  |  |  |  |  |
| **non-COPE** | N=2873 | N=2567 |  |  |  |  |
|  | LSM (SE) | LSM (SE) |  |  |  |  |
|  | 8.21 (0.02) | 8.24 (0.02) | 0.04 | 0, 0.08 | 0.04 | 0, 0.08 |
|  |  |  | *P* interaction | <0.0001 | *P* interaction | <0.0001 |
|  | **PRE** | **POST** | **LDL** | | | |
| **COPE** | N=60 | N=64 |  |  |  |  |
|  | LSM (SE) | LSM (SE) |  |  |  |  |
|  | 93.51 (4.30) | 83.36 (4.24) | -10.15 | -17.10, -3.20 | -9.07 | -16.03, -2.11 |
|  |  |  |  |  |  |  |
| **non-COPE** | N=1387 | N=1217 |  |  |  |  |
|  | LSM (SE) | LSM (SE) |  |  |  |  |
|  | 95.27 (2.10) | 90.90 (2.13) | -4.37 | -5.98, -2.76 | -3.73 | -5.34, -2.12 |
|  |  |  | *P* interaction | 0.11 | *P* interaction | 0.14 |
|  | **PRE** | **POST** | **SBP** | | | |
| **COPE** | N=113 | N=113 |  |  |  |  |
|  | LSM (SE) | LSM (SE) |  |  |  |  |
|  | 132.59 (1.10) | 134.03 (1.09) | 1.44 | 0.14, 2.73 | 1.25 | -0.06, 2.55 |
|  |  |  |  |  |  |  |
| **non-COPE** | N=1964 | N=1950 |  |  |  |  |
|  | LSM (SE) | LSM (SE) |  |  |  |  |
|  | 131.23 (0.49) | 132.37 (0.49) | 1.14 | 0.80, 1.47 | 0.9 | 0.56, 1.24 |
|  |  |  | *P* interaction | 0.66 | *P* interaction | 0.62 |
|  | **PRE** | **POST** | **BMI** | | | |
| **COPE** | N=59 | N=59 |  |  |  |  |
|  | LSM (SE) | LSM (SE) |  |  |  |  |
|  | 36.72 (0.73) | 36.23 (0.73) | -0.48 | -0.97, 0.002 | -0.36 | -0.85, 0.12 |
|  |  |  |  |  |  |  |
| **non-COPE** | N=1196 | N=1168 |  |  |  |  |
|  | LSM (SE) | LSM (SE) |  |  |  |  |
|  | 35.87 (0.18) | 35.49 (0.18) | -0.38 | -0.50, -0.25 | -0.26 | -0.38, -0.13 |
|  |  |  | *P* interaction | 0.68 | *P* interaction | 0.68 |

Abbreviations: COPE, Community Outreach and Patient Empowerment; HbA1c, glycosylated hemoglobin; LDL, low-density lipoprotein (mg/dl); SBP, systolic blood pressure (mm Hg); BMI, body mass index (kg/m^2^); LSM, Least Squares Means; SE, Standard Error; CI, confidence interval; P interaction, p-value of the interaction for the β of the term intervention(0/1)*time (pre/post).

^a^ Model adjusted for the pre-1 years’ means.

^b^ Adjusted model for pre-1 years’ means, age (years; continuous), gender (male/female), preferred language (English, Navajo and other), primary care physician (yes/no); essential hypertension (yes/no), major depression disorder (yes/no), alcohol abuse (yes/no), dyslipidemia (yes/no), and major cardiovascular disease (yes/no).

**APPENDIX**

**Linear Mixed Models details:**

Y_ikj_ = β_0_ + β_1_TIME_ikj_ + β_2_COPE_ikj_ + β_3_COPE_ikj_*TIME_ikj_ + β_p_X_1ikj_ … + β_p_X_pikj_ + b_ik_ + s_k_ + ε_ikj_

In the model, Y_ikj_ is the outcome value for participant i at the visit date j in the site k, β_0_ is the overall intercept, b_ik_ is the separate random intercept for subject i at the site k, and s_k_ is a separate random intercept for site k. Random effects are assumed to follow a normal distribution. For participant i at the visit date j at site k, TIME_ikj_ (binary) indicates whether the outcome is measured Pre or Post enrollment; COPE_ikj_ (binary) indicates COPE versus non-COPE subjects; COPE_ikj_*TIME_ikj_ is the interaction term; X_1ikj_–X_pikj_ are the covariates. β_1_ represents the coefficient of time for a specific group (COPE or non-COPE); β_2_ represents the difference between COPE and non-COPE patients in the Pre-enrollment period; β_3_ represents the coefficient of different changes in outcome between COPE and non-COPE subjects between Pre and Post intervention.
